# Identification of point mutations and large intragenic deletions in Fanconi anemia using next‐generation sequencing technology

**DOI:** 10.1002/mgg3.160

**Published:** 2015-07-02

**Authors:** Elena Nicchia, Chiara Greco, Daniela De Rocco, Vanna Pecile, Angela D'Eustacchio, Enrico Cappelli, Paola Corti, Nicoletta Marra, Ugo Ramenghi, Marta Pillon, Piero Farruggia, Carlo Dufour, Alberto Pallavicini, Lucio Torelli, Anna Savoia

**Affiliations:** ^1^ Department of Medical SciencesUniversity of TriesteTriesteItaly; ^2^ Institute for Maternal and Child Health – IRCCS Burlo GarofoloTriesteItaly; ^3^ Clinical and Experimental Hematology UnitG. Gaslini Children's HospitalGenoaItaly; ^4^ Pediatrics UnitSan Gerardo HospitalMonzaItaly; ^5^ Pediatric Hematology UnitSantobono Pausilipon HospitalNaplesItaly; ^6^ Department of Pediatric and Public Health SciencesUniversity of TorinoTorinoItaly; ^7^ Pediatric Onco‐Haematology ClinicUniversity of PaduaPaduaItaly; ^8^ Pediatric Onco‐HematologyARNAS Civico HospitalPalermoItaly; ^9^ Department of Life SciencesUniversity of TriesteTriesteItaly; ^10^ Department of Mathematics and GeosciencesUniversity of TriesteTriesteItaly

**Keywords:** Fanconi anemia, next‐generation sequencing, ion PGM system, point mutations, copy number variations, diagnosis

## Abstract

Fanconi anemia (FA) is a rare bone marrow failure disorder characterized by clinical and genetic heterogeneity with at least 17 genes involved, which make molecular diagnosis complex and time‐consuming. Since next‐generation sequencing technologies could greatly improve the genetic testing in FA, we sequenced DNA samples with known and unknown mutant alleles using the Ion PGM ^™^ system (IPGM). The molecular target of 74.2 kb in size covered 96% of the FA‐coding exons and their flanking regions. Quality control testing revealed high coverage. Comparing the IPGM and Sanger sequencing output of *FANCA*,*FANCC*, and *FANCG* we found no false‐positive and a few false‐negative variants, which led to high sensitivity (95.58%) and specificity (100%) at least for these two most frequently mutated genes. The analysis also identified novel mutant alleles, including those in rare complementation groups *FANCF* and *FANCL*. Moreover, quantitative evaluation allowed us to characterize large intragenic deletions of *FANCA* and *FANCD2*, suggesting that IPGM is suitable for identification of not only point mutations but also copy number variations.

## Introduction

Fanconi anemia (FA) is an autosomal or X‐linked recessive bone marrow failure disease associated with congenital malformations and increased risk of developing hematological and solid tumors (Kee and D'Andrea 2012). FA is caused by mutations in at least 17 genes, (Bogliolo et al. 2013; Kottemann and Smogorzewska 2013; Sawyer et al. 2015), three [*FANCA* (OMIM 607139), *FANCC* (OMIM 613899), and *FANCG* (OMIM 602956)] of which account for ~80% of the FA cases. Except for a few founder effects in specific populations, there is a wide spectrum of private mutations, including large intragenic deletions (The Rockefeller University. Fanconi Anemia database, http://www.rockefeller.edu/fanconi/).

The genetic heterogeneity together with the numerous mutations that affect the FA genes makes molecular diagnosis complex. It is a tiered process starting from clinical suspicion that is then confirmed at the cellular level, testing sensitivity of patient's cells to DNA interstrand cross‐linking agents, such as diepoxybutane (DEB test) (Auerbach 1993). If the DEB test is positive, identification of mutations is fundamental for a correct management of patients in terms of genetic counseling, carrier testing, and prenatal diagnosis. An inconclusive DEB test might occur in patients with hematopoietic mosaicism arising from spontaneous genetic events, such as back mutations, leading to reversion of the FA cellular phenotype (Waisfisz et al. 1999; Gross et al. 2002).

Without any knowledge on candidate genes, molecular genetic testing usually starts from *FANCA*, which is mutated in 60–80% of FA families (Castella et al. 2011; De Rocco et al. 2014). The analysis consists of Sanger sequencing of 43 coding exons combined with MLPA (Multiple Ligation‐dependent Probe Amplification), being almost 20% of the *FANCA* alleles characterized by large intragenic deletions due to numerous *Alu* repeats localized within its intronic regions (Morgan et al. 1999). If no *FANCA* mutation is identified, the screening is extended to the other FA genes. Alternatively, the candidate gene can be identified by complementation analysis or restricted to components of the FA pathway playing a role up‐ or down‐stream of *FANCD2* (OMIM 613984) on the basis of its monoubiquitination status (Bogliolo et al. 2013; Kottemann and Smogorzewska 2013; Sawyer et al. 2015).

Whatever is the procedure, identifying FA mutations is expensive and time‐consuming. The analysis would greatly benefit from application of next‐generation sequencing (NGS) technologies, such as the Ion PGM^™^ (IPGM; Life Technologies) platform. This technology was applied to 28 FA patients and allowed us to identify 45 FA mutant alleles, including seven large intragenic deletions of *FANCA*, suggesting that the IPGM sequencer well responds to the requirements for the screening of the FA genes.

## Methods

### Biological samples

Thirty DNA samples (14 males and 16 females), 28 from FA probands with positive DEB test and two from healthy controls, were analyzed in this study (Table 1). All the subjects or their legal guardians gave written informed consent to the investigation, according to the Declaration of Helsinki. DNA samples were isolated from peripheral blood (*n* = 25) or from lymphoblastoid cell lines (*n* = 5) using a High Pure PCR Template Preparation Kit (Roche, Indianapolis, USA). In 25 samples, at least one of the most frequently mutated genes (*FANCA*,* FANCC* or *FANCG*) was previously analyzed by Sanger sequencing (De Rocco et al. 2014). Of the 28 affected individuals, both FA alleles were characterized in 15 cases. In three cases, including two with hematopoietic mosaicism (Waisfisz et al. 1999), only one FA allele was identified. In this cohort, 23 FA alleles were still missing. All samples were tested using the Salsa MLPA kit with probe mix P031/P032 according to the manufacture protocol (MRC‐Holland, Amsterdam, the Netherlands). Reactions were run on ABI PRISM 3130 Genetic Analyzer (Applied Biosystems, Foster City, CA), and analyzed by Gene Mapper version 3.2 and MRC‐Holland Coffalyzer software.

**Table 1:** Features of DNA samples included in the study

DNA sample ID	Gender	DNA sample source^a^	FANC genes analyzed by Sanger sequencing	Mutant gene	Mutations identified by Sanger sequencing^b^	Mutations identified by Ion PGM^TM^ b	Reference
W1	M	PBC	A, G	Wild type	None	None	This paper
W2	F	PBC	A, C, G	Wild type	None	[c.50C>G, p.(Pro17Arg) in *FANCL*]	This paper
P3	M	PBC	A	*FANCA*	c.3558dup p.(Arg1187Glufs*28)	Excluded from all analyses	De Rocco et al. (2014) (FA29)
					**c.1360‐?_2778+?del p.(Ala454_His926del)** **(del ex15_28)**		
P4	F	PBC	A	*FANCA*	**c.1360‐?_1470+?del** p.(Ala454_Gln490del) **(del ex15)**	Excluded from CNV analysis	De Rocco et al. (2014) (FA40)
					**c.894‐?_3348+?del** p.(Trp298*) **(del ex11_33)**		
P5	M	PBC	A	*FANCA*	c.3788_3790del p.(Phe1263del)	Confirmed	De Rocco et al. (2014) (FA1)
					c.3788_3790del p.(Phe1263del)		
P6	F	PBC	A	*FANCA*	c.3239G>A p.(Arg1080Gln)	Confirmed	De Rocco et al. (2014) (FA10)
					c.3971C>T p.(Pro1324Leu)		
P7	M	PBC	A	*FANCA*	c.457C>T p.(Gln153*)	Confirmed [c.1466T>C, p.(Ile489Thr) in *FANCJ* and c.1249G>T, p.(Glu417*) in *FANCM*]	De Rocco et al. (2014) (FA13)
					c.709+1G>A		
P8	M	PBC	A	*FANCA*	c.3788_3790del p.(Phe1263del)	Confirmed [c.1096_1099dup, p.(Thr367Asnfs*13) in *FANCL*]	De Rocco et al. (2014) (FA21)
					c.3971C>T p.(Pro1324Leu)		
P9	F	PBC	A	*FANCA*	c.2638C>T p.(Arg880*)	Confirmed [**c.‐78‐?_5024+?del (del ex1_44) in ** ***FANCD2***]	De Rocco et al. (2014) (FA37)
					c.3164G>A p.(Arg1055Gln)		
P10	F	LCL	A	*FANCA*	c.3638_3639del p.(Pro1213Argfs*64)	Confirmed	De Rocco et al. (2014) (FA42)
					c.3971C>T p.(Pro1324Leu)		
P11	F	LCL	A	*FANCA*	c.3761_3762dup p.(Glu1255Argfs*12)	False negative^c^	De Rocco et al. (2014) (FA43)
					c.2574C>G p.(Ser858Arg)	Confirmed [c.2204G>A, p.(Arg735Gln) in *FANCD2*]	
P12	M	PBC	A	*FANCA*	c.2290C>T p.(Arg764Trp)	Confirmed	De Rocco et al. (2014) (FA44)
					c.4029T>G p.(His1343Gln)		
P13^d^	M	PBC	A	*FANCA*	c.1450G>C p.(Glu484Gln)	Confirmed	This paper
					Not found	Not found	
P14	M	PBC	A, G	*FANCA*	c.1115_1118del p.(Val372Alafs*42)	Confirmed	De Rocco et al. (2014) (FA85)
					c.1126C>T p.(Gln376*)		
P15	M	PBC	A	*FANCA*	c.2812_2830dup p.(Asp944Glyfs*5)	Confirmed	De Rocco et al. (2014) (FA20)
					**c.‐42‐?_5481+?del (del ex1_43)**		
P16	M	PBC	A	*FANCA*	c.1850_1859del p.(Leu617Profs*20)	Confirmed	De Rocco et al. (2014) (FA67)
					**c.1827‐?_2778+?del** p.(Arg609Serfs*2) **(del ex21_28)**		
P17	F	PBC	A	*FANCA*	c.457C>G p.(Gln153Glu)	Confirmed [c.2816T>G, p.(Leu939Trp) in *FANCN*]	De Rocco et al. (2014) (FA65)
					c.3490C>T p.(Pro1164Ser)		
					**c.1471‐?_1626+?del** p.(Val491_Glu542del) **(del ex16_17)**		
P18	F	PBC	A, C, G	*FANCA*	c.893+5G>A p.(Phe879Valfs*19)	Confirmed [c.1151C>T, p.(Ser384Phe) in *FANCD1*]	De Rocco et al. (2014) (FA28)
					**c.190‐?_283+?del** p.(Val64Alafs*43) **(del ex3)**		
P19^e^	F	PBC	A, G	*FANCA*	**c.1360‐?_1826+?del** p.(Ala454Serfs*3) **(del ex15_20)**	Confirmed	De Rocco et al. (2014) (FA61)
					Not found	Not found	
P20^f^	M	PBC	A, C, G	*FANCA*	c.4258G>T p.(Glu1420*)	Confirmed [c.874C>G, p.(Pro292Ala) and c.5848T>G, p.(Leu1950Val) in *FANCM*]	De Rocco et al. (2014) (FA41)
					Not found	Not found	
P21	F	PBC	A, G	*FANCC*	Not found	c.67del p.(Asp23Ilefs*23)	De Rocco et al. (2014) (FA88)
						c.67del p.(Asp23Ilefs*23)	
P22	F	LCL	A, G	*FANCC*	Not found	c.37C>T p.(Gln13*)	De Rocco et al. (2014) (FA89)
						c.692_694del p.(Lys231del)	
P23	F	PBC	A, G	*FANCC*	Not found	c.37C>T p.(Gln13*)	This paper
						c.1069C>T p.(Gln357*) [c.9875C>T, p.(Pro3292Leu) in *FANCD1*]	
P24	F	LCL	G	*FANCF*	Not found	c.484_485del p.(Leu162Aspfs*103)	This paper
						c.484_485del p.(Leu162Aspfs*103)	
P25	M	PBC	A, G	Not found	Not found	Not found	This paper
P26	M	PBC	None	*FANCA*	nd	c.3788_3790del p.(Phe1263del)	De Rocco et al. (2014) (FA58)
						c.826+3del p.(250_251insGlyAlaPhe MetThrArgCysGlyPheLeu)	
P27	F	PBC	None	*FANCA*	nd	c.1776+7A>G p.(Ile573Serfs*12)	De Rocco et al. (2014) (FA66)
						**c.1827‐?_2852+?del** p.(Ala610_Arg951del) **(del ex21_29)**	
P28	F	PBC	None	*FANCA*	nd	c.548G>A p.(Trp183*)^g^	De Rocco et al. (2014) (FA49)
						**c.523‐?_2981+?del** p.(Ser175Leufs*5) **(del ex6_30)**	
P29	F	PBC	None	*FANCA*	nd	c.3660del p.(Asn1221Thrfs*26)	De Rocco et al. (2014) (FA47)
						c.50dup p.(Arg18Profs*19)^g^	
P30	M	LCL	None	*FANCL*	nd	c.50C>G p.(Pro17Arg)	This paper
						c.676C>T p.(Arg226Cys)	
						c.1021T>A p.(Trp341Arg)	

a PBC, peripheral blood cells; LCL, lymphoblast cell line.

b Nucleotide A of the ATG translation initiation start site of the *FANCA, FANCC, FANCD1, FANCD2, FANCF, FANCG, FANCJ, FANCL, FANCM,* and *FANCN* cDNAs from GenBank sequences NM_000135.2, NM_000136.2, NM_000059.3, NM_001018115.1, NM_022725.3, NM_004629.1, NM_032043.2, NM_018062.3, NM_020937.2, and NM_024675.3, respectively is indicated as nucleotide +1. In square brackets are potential pathogenetic heterozygous variants identified in genes different from that causing the disease. Large genomic deletions are indicated in bold.

c Mutation localized in a region where the reverse and forward primers of two adjacent amplicons aligned, reducing sequence efficiency of this mutant allele. Mutation was not called by the TSVC because seen only 47 of 262 reads.

d Sib of patient FA70 in De Rocco et al. (2014). As FA 70, who was found to be a mosaic as the second mutant allele (c.596+1G>T) was identified in fibroblast cells but not in LCL, P19 was enrolled as an FA patient with a potential hematopoietic mosaicism. For this reason, the second mutant allele could be missed.

e Patient with potential hematopoietic mosaicism because of revertant lymphoblastoid cell line. Complementation analysis carried out in peripheral blood T lymphocytes assigned this patient to *FANCA* genetic group but only one heterozygous mutation was identified. Due to the mosaic suspicion, the second mutant allele could be missed.

f Potential hematopoietic mosaicism status was not ascertained in this patient, whose peripheral blood cells were the only biological sample available.

g Identified by Sanger sequencing of uncovered amplicons.

### IPGM sequencing

The IPGM sequencing was carried out according to the manufacture's protocols (Life Technologies, Carlsbad, CA). The following FA genes were targeted: *FANCA* (GenBank NM_000135.2), *FANCB* (OMIM 300515; GenBank NM_001018113.1), *FANCC* (GenBank NM_000136.2), *BRCA2* (*FANCD1*; OMIM 600185; GenBank NM_000059.3), *FANCD2* (GenBank NM_001018115.1), *FANCE* (OMIM 613976; GenBank NM_021922.2), *FANCF* (OMIM 613897; GenBank NM_022725.3), *FANCG* (GenBank NM_004629.1), *FANCI* (OMIM 611360; GenBank NM_001113378.1), *BRIP1* (*FANCJ*; OMIM 605882; GenBank NM_032043.2), *FANCL* (OMIM 608111; GenBank NM_018062.3), *FANCM* (OMIM 609644; GenBank NM_020937.2), *PALB2* (*FANCN*; OMIM 610355; GenBank NM_024675.3), *RAD51C* (*FANCO*; OMIM 602774; GenBank NM_058216.2), *SLX4 (FANCP*; OMIM 613278; GenBank NM_032444.2), *ERCC4* (*FANCQ*; OMIM 133520; GenBank XM_011522424.1). The *BRCA1* (*FANCS*; OMIM 113705) was not included in the analysis because of its recent identification (Sawyer et al. 2015).

Briefly, to sequence the coding exons and their flanking regions of the 16 FA genes primers were designed using the Ion Ampliseq Designer software (Life Technologies, Carlsbad, CA, https://www.ampliseq.com/browse.action) and divided in two pools. Libraries were prepared using Ion Ampliseq library kit 2.0. Template were enriched in the Ion One Touch 2 system (Ion PGM template OT2 200 kit) and sequenced on IPGM sequencer (IPGM Sequencing 200 Kit v2). In order to assure high coverage, 12 libraries (6 DNA samples) were loaded into 316 chips. For quality evaluation and alignment to the hg19 human genomic sequence, sequencing data were analyzed using the Ion Torrent Suite software's plug‐ins (Lifetechnologies, Carlsbad, CA) Coverage Analysis (TSCA, v4.0) and Variant caller (TSVC, v.4.0). Functional annotation was performed using the w‐annovar software (http://wannovar.usc.edu/). Libraries alignment was visualized using Integrative Genomics Viewer (IGV) software (Thorvaldsdóttir et al. 2013). To confirm variants, Sanger sequencing was carried out on FA exons as previously described (De Rocco et al. 2014).

### Detection of CNV

Statistical analyses for detection of copy number variations (CNV) implied two normalizations for each sample. The first was an intrasample normalization, which was calculated by dividing the total number of reads of each amplicon by the total reads average obtained from the same library. Since almost 20% of the *FANCA* alleles are large intragenic deletions and *FANCB* is localized on chromosome X (Morgan et al. 1999), we excluded the *FANCA* and *FANCB* amplicons from the calculation of the total reads of each library. The second was an intersample normalization, which was determined by dividing the intrasample normalization of each amplicon by the average of all the intrasample normalization of this amplicon from control samples. As control samples in the intersample normalization of *FANCA* and *FANCB* amplicons, we used data from 21 samples without *FANCA* deletions (from MLPA analysis) and from 15 females, respectively. For the intrasample normalization of amplicons of the other FA genes, we use all the samples (*n* = 28) except for P3 and P4, which had the lowest uniformity amplicon coverage. All those amplicons that did not achieve the threshold of 30× in more than three samples were excluded from the analysis.

We considered intersample normalization ratios lower than 0.7 indicative of heterozygous deletions and ratios higher than 1.3 suggestive of duplications. Graphs were performed using the software Minitab^®^ Statistical Software (version 16, Minitab INC., State College, Pennsylvania). Statistical analyses were performed using the statistical software Minitab (version 16). Median and interquartile range (IQR) were used to described nonnormal distribution data, whose comparison was performed by the Mann–Whitney test.

## Results

### IPGM sequencing quality: high coverage for 95% of amplicons

The FA genes represented a target of 74.2 kb split into 693 amplicons ranging in size from 125 to 225 bp. The amplicons cover 96% of the coding exon sequences with ~30 bp of their flanking intronic sequences. The regions excluded by the design (4%) are variably dispersed through all the genes, varying from 0.03% (*FANCQ*) to 10% (*FANCE*) of the targeted sequences (data not shown). All samples satisfied the quality control parameters except for P3, a partially degraded DNA that was excluded from further analysis because the uniformity of amplicon coverage was below 50% (Table S1).

Coverage analysis found that 35 of the 693 amplicons did not reach the threshold of 30× in at least one of the samples (Table S2). Five amplicons were not covered in at least 10 samples, suggesting that “constitutive” features of DNA could be responsible for the low sequencing efficiency. It is reasonable to hypothesize that repetitive sequences or primer self‐annealing interfere with amplification rate. For instance amplicon AMPL544050257 (exon 1 of *FANCA*), which is a GC‐rich region (78%), did not reach the threshold of 30× in 15 samples. For amplicons AMPL1197148282 (exon 4 of *FANCB*), AMPL404630568 and AMPL388340296 (exons 11 and 20 of *FANCD1*), and AMPL433543078 (exon 17 of *FANCJ*) the coverage was less than 30× in 12, 10, 21, 15 samples, respectively, more likely because of self‐annealing or intra‐strand loop formation of primers, as revealed by inspection of primer sequences. For the remaining 30 amplicons the coverage was less than 30× in 1–7 samples, indicating the presence of “occasional” interfering factors (Table S2). The two amplicons covering exon 15 of *FANCA* were not sequenced in P4, which carried two large heterozygous deletions encompassing this exon (Table 1). In P28, a compound heterozygote for the c.523‐?_2981+?del and c.548G>A mutations of *FANCA*, amplicon AMPL544193477 (exon 6) was not covered because deleted on one allele and not amplified on the other for the presence of c.548G>A in one of the primer complementary sequence. Inspection of the other uncovered amplicons did not provide us with any insight into the potential mechanisms of their low coverage. It is likely that allelic drop‐out for the presence of rare variants is involved.

### High sensitivity and specificity of IPGM sequencing for *FANCA*,* FANCC*, and *FANCG*

**Figure 1:**
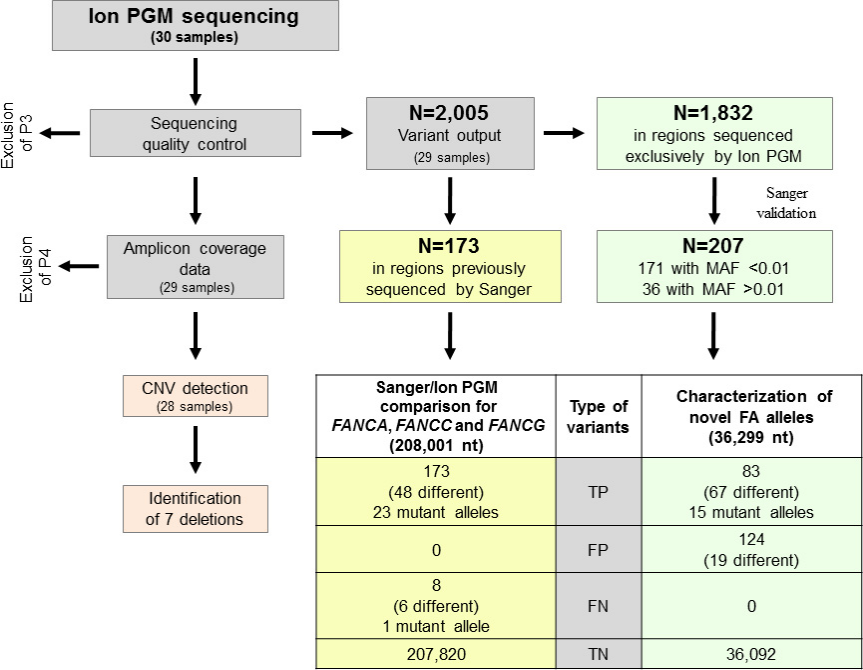
Schematic representation of the study. The IPGM sequencing output underwent a quality control process and analysis of the amplicon coverage data to detect CNVs. Of the 2005 variants called by IPGM, 173 were localized in regions (208,001 nt) of *FANCA*,*FANCC*, and *FANCG* that were previously analyzed by Sanger sequencing. All were TP. Although no FP variants were called, IPGM did not detected eight FN. Of the remaining 1832 variants, only those (*N* = 171) with a MAF < 0.01 were taken into consideration. Sanger sequencing of regions containing these variants tested another 36 variants (*N* = 207 total variants) and identified 83 TP and 124 FP. IPGM, Ion PGM ^™^; CNV, copy number variations; TP, true positive; FP, false‐positive; FN, false negative; MAF, minor allele frequency; TN, true negative.

After sequencing quality evaluation, we determined whether the variants called in the 29 samples were true (TPs) or false positives (FPs). Of the 2005 annotations (~70 per sample), we selected those (*n* = 173; 48 different) that were located in the genomic regions of the *FANCA*,* FANCC*, and *FANCG* genes that were previously screened by Sanger sequencing (208,001 nt) (Fig. 1). Comparing data from the two technologies, we found that all the 173 variants were TPs because also detected by Sanger sequencing. Among these, 23 were known mutations of *FANCA* (Table 1). Of note, no FPs was annotated. In the same regions, however, the IPGM sequencing did not call eight false‐negative variants (FNs; 6 different), one of which was the heterozygous c.3761_3762dup mutation (*FANCA*) identified in sample P11. This duplication was likely to have not been called because of allelic drop‐out being located in exon 37 where the reverse and forward primers of the two adjacent amplicons aligned.

Using these data (TP = 173, FP = 0, and FN = 8) and considering the number of true negative (TN; *N* = 207,820), we calculated sensitivity [TP/(TP + FN)] and specificity [TN/(TN + FP)] that, at least for the FANCA, FANCC, and FANCG genes, were 95.58% and 100%, respectively (Fig. 1).

### Characterization of novel FA alleles

In addition to these 173 TPs, the IPGM sequencing called another 1832 variants (Fig. 1), some of which were expected to be the mutations yet uncharacterized in our cohort (23 alleles) (Table 1). We selected those (*N* = 171) with minor allele frequency (MAF) <0.01, as derived from dbSNP database. Screening the genomic regions containing these variants by Sanger sequencing (36,299 nt; Fig. 1), we could analyze 36 additional annotations that were previously excluded because of their MAF > 0.01. Of these 207 variants, 83 (67 different) were TPs and 124 (19 different) FPs.

Among the 67 different TPs (Table S3), we searched for known FA mutations and/or deleterious variants, such as nonsense and frameshift mutations, amino acid substitutions with high pathogenetic prediction, and putative alternative splicing variants (Table S3). This allowed us to characterize 13 mutant alleles of *FANCA*,* FANCC*, and *FANCF* in eight (P21, P22, P23, P24, P26, P27, P28, and P29) of the 10 samples without any FA mutations (Table 1). In sample P30, we identified three different missense mutations (p.Pro17Arg, p.Arg226Cys, and p.Trp341Arg) in *FANCL*, whose pathogenetic effect has been demonstrated by complementation analysis (De Rocco and Hanenberg, unpubl. ms.). Of interest, one heterozygous pathogenetic variant of *FANCL* (p.Pro17Arg) was also confirmed by Sanger sequencing in control sample W2 (Table 1). The remaining TPs were heterozygous variants, most of which reported in the dbSNP database. They mainly affect intronic regions outside the consensus splice site sequences, though five were expected to create cryptic splice sites (Table S3). Others were synonymous or benign missense variants, as determined by bioinformatics tools. However, nine were expected to be pathogenetic. They were seven missense mutations of *FANCD1*,* FANCD2*,* FANCJ*,* FANCM,* and *FANCN*, one frameshift (p.Thr367Asnfs*13) and one nonsense (p.Glu417*) mutations of *FANCL* and *FANCM*, respectively (Tables 1; S3).

In this analysis, 124 FPs were also annotated (Table S4). Most of the 19 different FPs were localized in homopolymeric regions (Bragg et al. 2013). Of note, four were called in more than 22 patients, suggesting that they could be filtered in a standardized diagnostic workflow.

### Identification of FA CNV: correct assignment of gender

**Figure 2:**
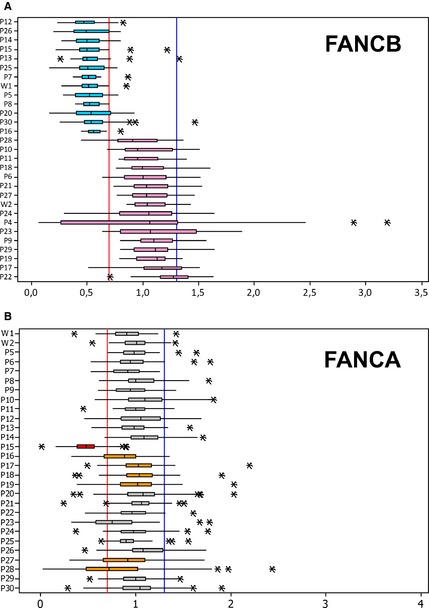
CNV analysis of the (A) *FANCB* and (B) *FANCA* amplicons. The values were represented by medians of intersample normalization ratio and box plots reporting the IQR. A median below 0.7 or between 0.7 and 1.3 is indicative of a single or double, respectively copy of the gene. In the *FANCB* box plot, the median is always below 0.7 and between 0.7 and 1.3 in all the males (blue) and females (pink), respectively. Even the IQR respects the thresholds except for female samples P4, P23, P17, and P22. Whereas in P23, P17, and P22 the 3rd quartiles are just above 1.3 (1.48, 1.34, and 1.41, respectively), in P4 the IQR ranges from 0.27 to 1.31. For this reason and for its low uniformity index (Table S1), P4 was excluded from CNV analysis. As determined by the Mann–Whitney test, the differences observed between males and females were not due to random variations of the *FANCB* coverage (Table S5). In the *FANCA* box plot, the mean is between 0.7 and 1.3 in all but one (P15) the samples. The 1st quartile was below 0.7 in P16 (0.67), P23 (0.58), P27 (0.66), and P28 (0.48), suggesting potential intragenic deletions of *FANCA*. The asterisks indicate the outliers. IQR, interquartile range; CNV, copy number variations.

To understand whether the IPGM data could be used for CNVs detection, we determined whether the analysis of *FANCB*, the only FA gene located on chromosome X, could allow us to distinguish male from female individuals. Consistent with our knowledge on gender, all males had median and IQR below 0.7, which is indicative of hemizygosity condition (Fig. 2). In females, whereas the median always ranged between 0.7 and 1.3, the box plots extended out of the normal range in four cases (P4, P17, P22, and P23). Whereas in P17, P22, and P23 the upper value of IQR was just above the threshold of 1.3, in P4 the attribution of gender was instead ambiguous as the IQR ranged from 0.27 to 1.31 (Fig. 2). Although we cannot exclude a partial deletion of *FANCB*, sample P4 was excluded from the CNV analysis because of its relatively low uniformity index (Table S1). The differences observed between males and females were not due to random variations of the *FANCB* coverage in almost all cases (Table S5).

### Deletions of the entire *FANCA* and *FANCD2* genes

Using the same criteria as above, we determined whether other FA genes were affected by CNVs. We found that the median and IQR of the intersample normalization ratios in *FANCA* and *FANCD2* was significant lower than expected in samples P15 and P9, respectively (Figs. 2 and S1). The *FANCA* and *FANCD2* deletions were confirmed by MLPA and SNP array, respectively, showing that the both hemizygous condition was extended to the entire gene (Figs. S2 and S3). Of note, since P9 was a compound heterozygous with two mutant alleles of *FANCA* (Table 1), it would be interesting to explore whether loss of function of *FANCA* together with haploinsufficiency of *FANCD2* makes the phenotype worse. However, clinical data from this affected individual are limited for any speculation.

### Characterization of large intragenic deletions of *FANCA*

**Figure 3:**
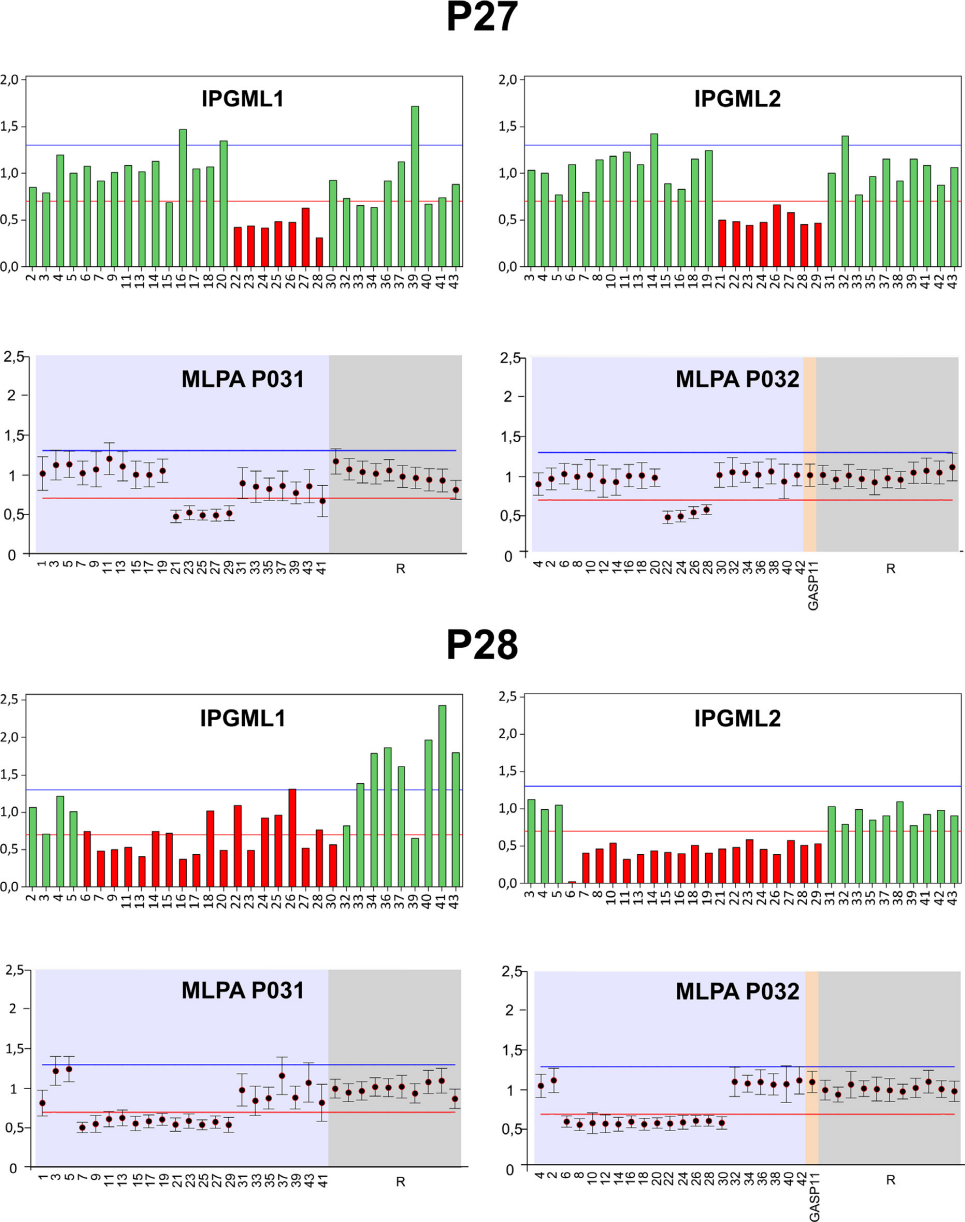
Detection of large intragenic deletions in *FANCA*. IPGM and MLPA analysis of two novel deletions of exons 21–29 (P27) and exons 6–30 (P28). Amplicons from the two IPGML1 and IPGML2 are reported in graphs showing hemizygous amplicons in red. MLPA output of two probes mix (MLPAP031 and MLPAP032) from the Coffalayzer.net software, showing *FANCA* exons and reference loci (R) values. In both IPGM and MLPA analysis, the intersample normalization of deleted adjacent exons is under the threshold of 0.7 (red line). IPGM, Ion PGM ^™^; MLPA, multiple ligation‐dependent probe amplification; IPGML, IPGM libraries.

In trying to detect partial deletions of *FANCA*, we first used the IGV software and found that in samples with known intragenic deletions the coverage of the deleted exons was reduced respect to that of the flanking undeleted amplicons (data not shown). Then, a statistical evaluation revealed that the first quartile of the coverage distribution were below the threshold of 0.7 in P16 (IQR = 0.67–1.0), P23 (IQR = 0.58–0.95), P27 (IQR = 0.66–1.09), and P28 (IQR = 0.48–1.01) (Fig. 2B). Considering that for small deletions the intersample normalization ratios of amplicon coverage is likely to reside within the normal range, we plotted the intersample normalization ratio of each amplicon. In P16, P17, P18, and P19 we confirmed the known CNVs (Table 1; Fig. S2). In P27 and P28 we identified two novel deletions encompassing exons 21–29 and exons 6–30, respectively, which were confirmed by MLPA analysis (Fig. 3). Despite its first quartile below threshold 0.7 (Fig. 2), amplicon plots and MLPA analysis did not confirm any CNV in P23 (data not shown). Since in P23 the first quartile was below the threshold even for other genes (Fig. S1), quality of this DNA sample could have interfered with the coverage efficiency.

### Uncharacterized FA alleles

Of the 23 FA alleles yet unknown in our cohort, the NGS identified 14 point mutations and two large genomic deletions. In order to characterize the seven missing variants, we carried out Sanger sequencing of all the uncovered amplicons in P13, P19, P20, P25, P28, and P29 (Table S1), allowing us to identify the second mutant *FANCA* allele, c.548G>A (exon 6) and c.50dup (exon 1), in P28 and P29, respectively. In the remaining patients, one (P13, P19, and P20) or two (P25) pathogenetic alleles remained uncharacterized, suggesting that mutations outside of the target design or affecting novel FA genes are involved (Castella et al. 2011).

## Discussion

The extensive allelic and nonallelic genetic heterogeneity of FA is one of the major problems in molecular diagnostic testing of the disease. In recent years, various strategies, mainly based on extensive screening of the FA genes or complementation or protein analyses, have been developed to optimize mutation detection rate (Castella et al. 2011; De Rocco et al. 2014). In order to improve and speed up this process, we evaluated the possibility to apply NGS strategies to identify both point and CNV alterations. The disease is caused by at least 17 different genes, which explain FA in almost all the affected individuals (Bogliolo et al. 2013; Kottemann and Smogorzewska 2013; Sawyer et al. 2015). Therefore, since only a small portion of FA patients are likely to have mutations in genes yet uncharacterized, we choose IPGM, a benchtop sequencer for targeted sequencing that has successfully been applied for mutational screening in tumors and other monogenic diseases (Anasagasti et al. 2013; Balla et al. 2014; Gu et al. 2014).

The 693 amplicons selected by the primer design software covered 96% of the targeted regions consisting of exons with their flanking regions of the FA genes. Sequencing quality was relatively high with most of the amplicons covered >30×. The uniformity values ranged from 87% to 98% in all but one (P3) the samples. Only a relatively limited number (*n* = 35) of amplicons did not reach the coverage threshold of 30× in a variable number (from 1 to 21) of samples. Whereas in P3 the low uniformity rate was likely to be due to low DNA quality, in the other cases nucleotide inspection revealed high CG content of amplicons, sequences favoring dimer and hairpin formation within oligonucleotides or SNPs leading to allelic drop‐out. However, the uncovered amplicons represented only 1% of the target regions, confirming high efficiency of the primer design software. It is plausible that designing new oligonucleotides, we would further reduce the percentage of regions with low coverage.

Considering all the samples, the IPGM output called 2005 variants. Taking advantage of our previous Sanger analyses (De Rocco et al. 2014), we compared the data output from the same genomic regions of *FANCA*,* FANCC*, and *FANCG* screened by the two techniques. In these regions, all the variants annotated by IPGM (*n* = 173 TPs) had also been detected by Sanger sequencing. Among these variants were the mutations accounting for all but one the known FA alleles. The undetected mutant allele (c.3761_3762dup in *FANCA* of P11) was one of the eight FNs that was not called because of allelic drop‐out. Inspection of the other few FNs revealed that they were mainly localized at the 5′ and 3′ ends of amplicons, where the coverage drops reducing sequencing reliability. If this takes place outside splice site regions, the probability to miss pathogenetic mutations is strongly reduced. However, amplicons are ~100 bp in size and larger exons are split into two or more amplicons with primers designed in coding regions that may be hit by mutations. If this occurs, as for c.3761_3762dup, pathogenetic variants could be missed. However, since we did not reveal any FPs, sensitivity and specificity was 95.58% and 100%, respectively at least for the analysis of *FANCA*,* FANCC*, and *FANCG*.

Among the other 1832 variants, we search for pathogenetic alterations to identify novel FA alleles. Since mutations are rare variants, we selected those with MAF < 0.01 (*n* = 171) for Sanger confirmation. Sequencing the relative exons, we could check another 36 variants with MAF > 0.01. Of the 207 variants, 83 were TPs and 124 FPs. No FN emerged from this analysis, which covered more than 35,000 nt spread through all the FA genes of several individuals. As expected (Bragg et al. 2013), the FPs were called in homopolymeric regions and some of them were recurrent in many samples, suggesting that some genomic regions are prone to FPs calling. Therefore, it is of fundamental importance to annotate at least the most frequent FPs for their systematic filtering.

Application of NGS is usually combined with MLPA or microarray techniques in a comprehensive approach for mutational screening. Large intragenic deletions are relatively common in FA, as the *FANCA* locus has high content of *Alu* repeats that could generate intragenic CNVs through unequal crossing‐over (Morgan et al. 1999). Indeed, massive parallel sequencing complemented with array comparative genomic hybridization and RNA sequencing analysis allowed the identification of biallelic germline mutations in different FA families (Chandrasekharappa et al. 2013). However, it has been shown that massively parallel sequencing techniques are feasible to identify CNV in FA and other diseases (Ameziane et al. 2012; Grasso et al. 2015). Similarly, we used the amplicon coverage data from IPGM to detect CNVs. First at all, we discriminated between males and females using the coverage data of *FANCB*, the only FA gene localized on chromosome X. Then, we confirmed five large deletions of *FANCA* we previously identified by MLPA (De Rocco et al. 2014). Moreover, we detected two novel heterozygous large deletions of *FANCA* in patients P27 and P28, which together with c.1776+7A>G and c.548G>A defined the *FANCA* alleles in these two patients. Finally, as confirmed by SNP array the entire *FANCD2* gene was deleted in P9, a patient with biallelic mutations in *FANCA*.

Targeting all the FA genes, we might identify mutations in more than one gene, as it occurred for P9 and another seven patients. Whenever the number of patients is large enough, it will be interesting to determine whether mutations in more FA genes associate with more severe phenotypes. Moreover, special attention should be paid to variants of *FANCM*. Although is not regarded anymore as an FA gene, it is a susceptibility gene for breast cancer and individuals (P9 and P20) carrying some of its variants could be at risk of developing this malignancy (Kiiski et al. 2014; Lim et al. 2014). Identification of mutations in different genes could unveil puzzling questions about correct assignment of the complementation group. The recessive model of inheritance or complementation analysis will help defining correctly the gene responsible for the disease.

In summary, the IPGM technology allowed us to obtain high‐quality sequencing data. In 19 samples, both mutant alleles were detected, including those in genes that, such as *FANCF* and *FANCL*, are only rarely taken into consideration in standard molecular diagnosis process. Only one heterozygous mutation was instead revealed in six individuals. In three of these patients, the second allele was missed because located in a CG‐rich region (P29) or because of allelic drop‐out (P11 and P28). Considering that another two (P13 and P19) were referred as potential hematopoietic mosaics and that a mosaic condition could not be excluded in P20, back mutations could have interfered with the detection of the second mutation if present only in a small percentage of reads. In these cases, DNA from different sources, such as from primary skin fibroblast, would be useful for comprehensive molecular genetic testing. Finally, in the last sample (P25) we did not find any mutation; it is likely that a novel FA gene is responsible for the disease in this patient. Taken together these data indicate that IPGM is a feasible platform for identifying mutations in the FA genes according to a flow chart arisen from this study (Fig. S4).

## Conflict of Interest

The authors declare no competing financial interests. MINITAB^®^ and all other trademarks and logos for the Company's products and services are the exclusive property of Minitab Inc. All other marks referenced remain the property of their respective owners. See minitab.com for more information.

## Supporting information


**Table S1.** Quality control data of 30 samples according to IPGM analysis.
**Table S2.** Amplicons not covered more than 30×.
**Table S3.** True positive (TP) variants identified during characterization of novel FA alleles.
**Table S4.** False positive (FP) variants identified during characterization of novel FA alleles.
**Table S5.** Mann‐Whitney test between males and females.
**Figure S1.** CNV analysis of the FA genes. The analysis is shown for all the FA genes except for *FANCA* and *FANCB* (Fig. 1) in 28 of the 30 samples included in this study (samples P3 and P24 have been excluded for their low amplicon uniformity). Box plots report median, interquartile range (IQR) and outliers (asterisks). A median below 0.7 or between 0.7 and 1.3 (red and blue vertical lines) is indicative of one or two copies, respectively of the gene. The median is in the normal range for all samples except for P9 in *FANCD2* and P23 in both *FANCE* and *FANCP*. The 1st and the 3rd quartiles are low and above the threshold of 0.7 and 1.3 in several samples, but for most of which with only slight deviations. Of note, *FANCE* and *FANCF*, the two genes covered by the lowest number of amplicons (18 and 8, respectively), show IQR deviations in a higher number of samples. Since in sample P23 both median and IQR were outside the normal ranges in eight genes without knowing the reasons, the deviations were not considered reliable enough for suspecting any CNV.
**Figure S2.** Detection of large intragenic known deletions of *FANCA*. IPGM and MLPA analysis showing deletions of the entire gene (P15), exons 21–28 (P16), exons 16–17 (P17), exons 3 (P18), and exons 15–20 (P19). Amplicons from the two IPGM libraries (IPGML1 and IPGML2) are reported in graphs showing hemizygous amplicons in red. MLPA output of two probes mix (MLPAP031 and MLPAP032) from the Coffalayzer.net software, showing *FANCA* exons and reference loci (R) values. In both IPGM and MLPA analysis, the intersample normalization of deleted adjacent exons is under the threshold of 0.7 (red line). Of note, in graph MLPAP031 of P15, the apparent homozygous deletion of exon 29 is a false positive of MLPA because the mutation on the second allele (c.2812_2830dup) is localized within the probe annealing sequence of exon 29.
**Figure S3.** Graphical output for copy number using SNP‐based arrays on chromosome 3 in P9. The plot for the B allele frequency shows a heterozygous deletion of 11.6 Mb (235,748–11,880,816) on chromosome 3p26.3‐p25.2 containing the *FANCD2* gene. In this patient the plot has 10% heterozygous (AB) SNP calls, as shown by the additional allele frequency, indicating a mosaicism of ~90%. For probes that are normal copy number, the signal intensity ratio of the subject versus controls is expected to be 1, and log_2_ R ratio should be ~0.0 (log_2_1 = 0). In the other plot loss of copy number results in a negative log_2_ ratio of ~−0.5. SNP array analysis was performed using the Human OmniExpress‐12 Bead Chip (Illumina Inc., San Diego, CA) according to Illumina's Infinium HD Assay protocol. Normalization of raw image intensity data, genotype clustering and individual sample genotype calls were performed using Illumina's GenomeStudio software v2011.1 (cnv partition 3.2.0). The CNV calls were determined with generalized genotyping methods implemented in the Penn CNV program.
**Figure S4.** Schematic representation of the molecular diagnostic workflow that can be applied in FA using the IPGM sequencing.
