# Supplementary material for: Identification of point mutations and large intragenic deletions in Fanconi anemia using next‐generation sequencing technology
Source: Mol Genet Genomic Med. 2015 Jul 2;3(6):500–12. doi: 10.1002/mgg3.160 (PMC4694132; doi:10.1002/mgg3.160)
Supplement: Supplementary file 1 — Table S1. Quality control data of 30 samples according to IPGM analysis. Table S2. Amplicons not covered more than 30×. Table S3. True positive (TP) variants identified during characterization of novel FA alleles. Table S4. False positive (FP) variants identified during characterization of novel FA alleles. Table S5. Mann‐Whitney test between males and females. Figure S1. CNV analysis of the FA genes. The analysis is shown for all the FA genes except for FANCA and FANCB (Fig. 1) in 28 of the 30 samples included in this study (samples P3 and P24 have been excluded for their low amplicon uniformity). Box plots report median, interquartile range (IQR) and outliers (asterisks). A median below 0.7 or between 0.7 and 1.3 (red and blue vertical lines) is indicative of one or two copies, respectively of the gene. The median is in the normal range for all samples except for P9 in FANCD2 and P23 in both FANCE and FANCP. The 1st and the 3rd quartiles are low and above the threshold of 0.7 and 1.3 in several samples, but for most of which with only slight deviations. Of note, FANCE and FANCF, the two genes covered by the lowest number of amplicons (18 and 8, respectively), show IQR deviations in a higher number of samples. Since in sample P23 both median and IQR were outside the normal ranges in eight genes without knowing the reasons, the deviations were not considered reliable enough for suspecting any CNV. Figure S2. Detection of large intragenic known deletions of FANCA. IPGM and MLPA analysis showing deletions of the entire gene (P15), exons 21–28 (P16), exons 16–17 (P17), exons 3 (P18), and exons 15–20 (P19). Amplicons from the two IPGM libraries (IPGML1 and IPGML2) are reported in graphs showing hemizygous amplicons in red. MLPA output of two probes mix (MLPAP031 and MLPAP032) from the Coffalayzer.net software, showing FANCA exons and reference loci (R) values. In both IPGM and MLPA analysis, the intersample normalization of deleted adjacent exons is under the threshold [file MGG3-3-500-s001.docx]

**Supplemental data**

**Identification of point mutations and large intragenic deletions in Fanconi anemia using Next Generation Sequencing technology**

Elena Nicchia^1^, Chiara Greco^2^, Daniela De Rocco^1^, Vanna Pecile^2^, Angela D’Eustacchio^2^, Enrico Cappelli^3^, Paola Corti^4^, Nicoletta Marra^5^, Ugo Ramenghi^6^, Marta Pillon^7^, Piero Farruggia^8^, Carlo Dufour^3^, Alberto Pallavicini^9^, Lucio Torelli^10^, Anna Savoia^1,2^

^1^Department of Medical Sciences, University of Trieste, Trieste, Italy;

^2^Institute for Maternal and Child Health – IRCCS Burlo Garofolo, Trieste, Italy;

^3^Clinical and Experimental Hematology Unit, G. Gaslini Children’s Hospital, Genoa, Italy;

^4^Pediatrics Unit, San Gerardo Hospital, Monza, Italy;

^5^Pediatric Hematology Unit, Santobono Pausilipon Hospital, Naples, Italy.

^6^Department of Pediatric and Public Health Sciences, University of Torino

^7^Pediatric Onco-Haematology Clinic, University of Padua, Padua; Italy;

^8^Pediatric Onco-Hematology, ARNAS Civico Hospital, Palermo, Italy

^9^Department of Life Sciences, University of Trieste, Trieste, Italy;

^10^Department of Mathematics and Geosciences, University of Trieste, Trieste, Italy;

**Supplemental tables**

**Table 1S. Quality control data of 30 samples according to IPGM analysis.**

| Sample | N. of bases per sample* | Bases with Q≥ 20  (%)^┼^ | Mapped reads^ǂ^ | Reads on target  (%)^§^ | Average reads per amplicon^ǁ^ | Uniformity of amplicon coverage (%)^¶^ | Amplicons reading end-to-end  (%)^#^ |
| --- | --- | --- | --- | --- | --- | --- | --- |
| W1 | 108,116,974 | 89.6 | 781,876 | 97.4 | 1,099 | 96.8 | 71.4 |
| W2 | 75,262,526 | 80.9 | 603,475 | 95.0 | 828 | 97.3 | 29.7 |
| P3 | 35,785,078 | 82.7 | 267,284 | 97.4 | 375 | 49.2 | 18.8 |
| P4 | 119,723,202 | 82.7 | 895,474 | 97.6 | 1,261 | 86.9 | 36.1 |
| P5 | 70,204,308 | 85.2 | 521,101 | 96.1 | 722 | 96.5 | 52.7 |
| P6 | 46,370,665 | 87.9 | 332,419 | 97.0 | 465 | 95.2 | 66.4 |
| P7 | 91,330,404 | 83.2 | 680,112 | 97.1 | 953 | 96.5 | 38.1 |
| P8 | 114,403,621 | 85.5 | 856,185 | 94.9 | 1,172 | 96.8 | 51.1 |
| P9 | 78,309,348 | 90.1 | 564,085 | 97.5 | 793 | 97.9 | 77.1 |
| P10 | 39,666,030 | 83.0 | 304,766 | 95.7 | 421 | 95.4 | 42.9 |
| P11 | 50,060,326 | 80.6 | 403,472 | 93.6 | 546 | 97.3 | 29.4 |
| P12 | 49,900,000 | 89.6 | 370,530 | 95.1 | 509 | 96.3 | 75.5 |
| P13 | 96,878,877 | 80.8 | 784,389 | 95.5 | 1,082 | 94.2 | 29.3 |
| P14 | 64,792,636 | 84.6 | 490,221 | 93.4 | 660 | 96.5 | 45.6 |
| P15 | 170,110,091 | 90.1 | 1,226,673 | 98.5 | 1,743 | 93.5 | 72.6 |
| P16 | 61,705,451 | 86.5 | 468,489 | 92.1 | 623 | 97.7 | 68.4 |
| P17 | 81,994,349 | 84.8 | 603,842 | 96.8 | 843 | 96.8 | 52.0 |
| P18 | 80,418,943 | 84.7 | 607,565 | 94.4 | 827 | 97.8 | 44.6 |
| P19 | 47,553,650 | 80.3 | 384,555 | 94.4 | 525 | 96.8 | 26.7 |
| P20 | 23,598,575 | 87.7 | 170,190 | 97.1 | 238 | 96.1 | 67.8 |
| P21 | 84,534,746 | 84.8 | 634,677 | 91.2 | 835 | 97.3 | 48.3 |
| P22 | 14,437,622 | 82.7 | 112,325 | 95.8 | 155 | 97.6 | 40.7 |
| P23 | 127,535,800 | 85.7 | 914,955 | 97.3 | 1,284 | 96.3 | 59.6 |
| P24 | 30,683,292 | 84.9 | 225,630 | 96.6 | 314 | 96.7 | 54.3 |
| P25 | 57,285,000 | 84.8 | 423,405 | 96.0 | 587 | 96.7 | 53.4 |
| P26 | 74,338,167 | 85.0 | 560,816 | 95.7 | 774 | 96.0 | 46.9 |
| P27 | 46,719,940 | 80.8 | 377,211 | 95.9 | 523 | 97.3 | 28.4 |
| P28 | 56,726,182 | 87.9 | 407,532 | 96.7 | 967 | 95.5 | 74.3 |
| P29 | 78,923,835 | 81.2 | 631,032 | 95.6 | 873 | 98.0 | 30.7 |
| P30 | 43,969,366 | 85.0 | 334,231 | 94.0 | 453 | 96.8 | 45.2 |
| Min | 14,437,622 | 80.3 | 112,325 | 91.2 | 155 | 49.2 | 18.8 |
| Max | 170,110,091 | 90.1 | 1,226,673 | 98.5 | 1,743 | 98.0 | 77.1 |
| Mean | 68,751,834 | 84.8 | 518,053 | 95.7 | 748 | 94.7 | 49.3 |
| St.dev | 32,789,486 | 2.9 | 240,79 | 16.8 | 350 | 8.8 | 16.8 |
| Median | 64,792,636 | 84.8 | 490,221 | 95.9 | 748 | 96.7 | 47.6 |
| 1^st^ quartile | 46,719,940 | 82.7 | 370,530 | 94.9 | 512 | 96.0 | 36.6 |
| 3^rd^ quartile | 81,994,349 | 86.3 | 631,032 | 97.0 | 933 | 97.3 | 64.7 |

*Total number of filtered and trimmed reads independent of length post filtering bases per barcode.

^┼^The percentage of reads that have a predicted quality score of Q20 or better. A Q20 score is the predicted quality of a Phred-like score of 20 or better, or one error in 100 bp.

ǂTotal number of reads mapped to the reference.

^§^The percentage of reads mapped to any targeted region relative to all reads mapped to the reference.

^ǁ^The average number of reads assigned to amplicons. The average number of reads assigned to amplicons.

^¶^ The percentage of bases in all targeted regions covered by at least 0.2x the average base read depth.

^#^The percentage of all amplicons that were considered to have a sufficient proportion of assigned reads (70%) that covered the whole amplicon target from 'end-to-end'. To allow for error the effective ends of the amplicon region for read alignment are within 2 bases of the actual ends of the region.

**Table 2S. Amplicons not covered more than 30X**

| Gene | Exon | ID amplicons | N. of samples  with coverage <30X (sample) |
| --- | --- | --- | --- |
| *FANCA*  (NM_000135.2) | 1 | AMPL544050257 | 15 |
|  | 6 | AMPL544193477 | 1 (P28) |
|  | 7 | AMPL2671673445 | 2 |
|  | 12 | AMPL544284467 | 5 |
|  | 15 | AMPL544674964 | 1 (P4) |
|  |  | AMPL544681421 | 1 (P4) |
|  | 16 | AMPL544342332 | 1 |
| *FANCB*  (NM_001018113.1) | 3 | AMPL544417387 | 4 |
|  | 4 | AMPL1197148282 | 12 |
|  |  | AMPL544382746 | 1 |
| *FANCC*  (NM_000136.2) | 4 | AMPL592054836 | 5 |
| *FANCD1*  (NM_000059.3) | 8 | AMPL388297720 | 1 |
|  | 11 | AMPL388360704 | 1 |
|  |  | AMPL404630568 | 10 |
|  |  | AMPL658814386 | 2 |
|  | 16 | AMPL624416753 | 6 |
|  | 20 | AMPL388340296 | 21 |
| *FANCD2*  (NM_001018115.1) | 17 | AMPL434371586 | 4 |
|  | 26 | AMPL442020299 | 1 |
| *FANCE*  (NM_021922.2) | 10 | AMPL544603911 | 1 |
| *FANCG*  (NM_004629.1) | 6 | AMPL543852913 | 5 |
| *FANCI*  (NM_001113378.1) | 11 | AMPL1190859878 | 1 |
|  | 32 | AMPL544478748 | 2 |
| *FANCJ*  (NM_032043.2) | 8 | AMPL416725112 | 7 |
|  | 11 | AMPL414710255 | 6 |
|  | 15 | AMPL434475581 | 1 |
|  | 17 | AMPL433543078 | 15 |
| *FANCL*  (NM_018062.3) | 1 | AMPL543889772 | 1 |
| *FANCM*  (NM_020937.2) | 5 | AMPL1156281934 | 3 |
|  | 6 | AMPL544176673 | 2 |
|  | 14 | AMPL2595994868 | 1 |
|  | 18 | AMPL1104380167 | 1 |
|  | 19 | AMPL1103979554 | 6 |
|  | 19 | AMPL1157014550 | 1 |
| *FANCP*  (NM_032444.2) | 14 | AMPL544488815 | 2 |

**Table 3S. True positive (TP) variants identified during characterization of novel FA alleles**

| Number | Gene | TP variants^┼^ | rs number  (MAF in 1000 Genome databse) | Pathogenicity | N. of samples  with TP  (Sample with pathogenetic variant) |
| --- | --- | --- | --- | --- | --- |
| 1 | *FANCA*  (NM_000135.2) | c.548G>A p.(Trp183*) | nr | nonsense | 1 (P28) |
| 2 |  | c.826+3del  p.(250_251insGlyAla  PheMetThrArgCysGlyPheLeu) | nr | splicing  (in frame)^§^ | 1 (P26) |
| 3 |  | c.1776+7A>G p.(Ile573Serfs*12) | nr | splicing  (frameshift)^§^ | 1 (P27) |
| 4 |  | c.3660del p.(Asn1221Thrfs*26) | nr | small deletion (frameshift) | 1 (P29) |
| 5 |  | c.3788_3790del p.(Phe1263del) | nr | small deletion  (in frame) | 1 (P26) |
| 6 |  | c.4260+29T>C | rs1800359 (0.18) | nd | 1 |
| 7 |  | c.4332T>G  p.(=) | rs149531696 (nr) | synonym | 1 |
| 8 | *FANCB*  (NM_001018113.1) | c.147A>G  p.(=) | nr | synonym  (generation of donor splice site - score 68)^╪^ | 1 |
| 9 | *FANCC*  (NM_000136.2) | c.37C>T  p.(Gln13*) | rs121917784 (nr) | nonsense | 2 (P22, P23) |
| 10 |  | c.67del  p.(Asp23Ilefs*23) homozygous | rs104886459 (nr) | small deletion (frameshift) | 1 (P21) |
| 11 |  | c.554G>A p.(Arg185Gln) | rs370346767 (nr) | missense (1)^╪^ | 1 |
| 12 |  | c.692_694del p.(Lys231del) | rs3831244 (nr) | small deletion  (in frame) | 1 (P22) |
| 13 |  | c.816C>T  p.(=) | rs55719336 (<0.01) | synonym | 1 |
| 14 |  | c.1069C>T p.(Gln357*) | nr | nonsense | 1 (P23) |
| 15 | *FANCD1*  (NM_000059.3) | c.800G>A p.(Gly267Glu) | rs80359036 (nr) | missense (0)^╪^ | 1 |
| 16 |  | c.1114A>C p.(Asn372His) | rs144848  (0.24) | missense (2)^╪^ | 1 |
| 17 |  | c.1151C>T p.(Ser384Phe) | rs41293475 (nr) | missense (5)^╪^ | 1 (P18) |
| 18 |  | c.1938C>T  p.(=) | rs28897711 (<0.01) | synonym | 1 |
| 19 |  | c.4258G>T p.(Asp1420Tyr) | rs28897727 (<0.01) | missense (2) ^╪^ | 1 |
| 20 |  | c.8755-66T>C | rs4942486  (0.47) | nd | 1 |
| 21 |  | c.8953+98T>C | rs81002901 (<0.01) | nd | 1 |
| 22 |  | c.9875C>T p.(Pro3292Leu) | rs56121817 (<0.01) | missense (8)^╪^ | 1 (P23) |

| 23 | *FANCD2*  (NM_001018115.1) | c.65-18A>C | nr | Nd | 1 |
| --- | --- | --- | --- | --- | --- |
| 24 |  | c.784-27del | nr | nd (generation of acceptor splice site - score 68)^╪^ | 1 |
| 25 |  | c.1634A>G p.(Asn545Ser) | rs145522204 (<0.01) | missense (2)^╪^ | 2 |
| 26 |  | c.2021+31C>T | rs3864015 (nr) | nd | 11 |
| 27 |  | c.2103G>T  p.(=) | rs139033444 (nr) | synonym | 1 |
| 28 |  | c.2204G>A p.(Arg735Gln) | nr | missense (8)^╪^ | 1 (P11) |
| 29 |  | c.2269+15C>T | nr | nd | 1 |
| 30 |  | c.2606-40A>T | rs36075953 (<0.01) | nd | 1 |
| 31 |  | c.2977-39C>T | rs45622438 (<0.01) | nd | 1 |
| 32 |  | c.3336-74G>A | rs34197804 (<0.01) | nd | 1 |
| 33 | *FANCE*  (NM_021922.2) | c.1572G>A  p.(=) | rs115195341 (<0.01) | synonym | 1 |
| 34 | *FANCF*  (NM_022725.3) | c.484_485del p.(Leu162Aspfs*103)  homozygous | nr | small deletion (frameshift) | 1 (P24) |
| 35 |  | c.557C>T p.(Ala186Val) | rs113910234 (<0.01) | missense (0)^╪^ | 1 |
| 36 | *FANCG*  (NM_004629.1) | c.1538G>A p.(Arg513Gln) | rs17885240 (<0.01) | missense (0)^╪^ | 1 |
| 37 |  | c.1636+7A>G | rs587118 (0.25) | unknown | 1 |
| 38 | *FANCI*  (NM_001113378.1) | c.755+55G>A | rs183255195 (<0.01) | generation of potential donor splice site  (score 66)^╪^ | 1 |
| 39 |  | c.976-125 T>C | nr | unknown | 1 |
| 40 |  | c.1963G>A p.(Gly655Arg) | rs138026584 (nr) | missense (3)^╪^ | 2 |
| 41 |  | c.1992+10T>C | nr | unknown | 1 |
| 42 |  | c.2203A>G p.(Ile735Val) | nr | missense (0)^╪^ | 1 |
| 43 |  | c.2225G>C p.(Cys742Ser) | rs2283432 (0.29) | missense (1)^╪^ | 1 |
| 44 |  | c.2292-120T>C | rs11855524 (0.07) | unknown | 1 |
| 45 |  | c.2367G>T  p.(=) | rs11857960 (0.08) | synonym | 1 |
| 46 | *FANCJ*  (NM_032043.2) | c.1466T>C p.(Ile489Thr) | nr | missense (7)^╪^ | 1 (P7) |
| 47 |  | c.2286T>C  p.(=) | rs61754141 (<0.01) | synonym | 1 |
| 48 |  | c.2906-31A>G | nr | generation of potential acceptor splice site  (score71)^╪^ | 1 |
| 49 | *FANCL*  (NM_018062.3) | c.50C>G  p.(Pro17Arg) | nr | missense (8)^╪^ | 2 (W2, P30) |
| 50 |  | c.676C>T p.(Arg226Cys) | nr | missense (8)^╪^ | 1 (P30) |
| 51 |  | c.1021T>A p.(Trp341Arg) | nr | missense (8)^╪^ | 1 (P30) |
| 52 |  | c.1096_1099dup p.(Thr367Asnfs*13) | nr | smallinsertion (frameshift) | 1 (P8) |

| 53 | *FANCM*  (NM_020937.2) | c.171G>C p.(Leu57Phe) | rs142007602 (<0.01) | missense (4)^╪^ | 1 |
| --- | --- | --- | --- | --- | --- |
| 54 |  | c.874C>G p.(Pro292Ala) | rs142747831 (<0.01) | missense (6)^╪^ | 1 (P20) |
| 55 |  | c.1249G>T  p.(Glu417*) | nr | nonsense | 1 (P7) |
| 56 |  | c.1397-58T>G | rs11157433 (0.24) | generation of potential acceptor splice site  (score 77)^╪^ | 2 |
| 57 |  | c.1397-16_1397-14del | nr | unknown | 2 |
| 58 |  | c.5848T>G p.(Leu1950Val) | rs146436929 (<0.01) | missense (6)^╪^ | 1 (P20) |
| 59 | *FANCN*  (NM_024675.3) | c.2586+58C>T | rs249954 (0.34) | unknown | 1 |
| 60 |  | c.2586+81C>T | rs114710547 (<0.01) | unknown | 1 |
| 61 |  | c.2587-38C>G | rs180177119 (nr) | unknown | 1 |
| 62 |  | c.2816T>G p.(Leu939Trp) | rs45478192 (<0.01) | missense (8)^╪^ | 1 (P17) |
| 63 | *FANCO*  (NM_058216.2) | c.790G>A p.(Gly264Ser) | rs147241704 (nr) | missense (3)^╪^ | 1 |
| 64 | *FANCP*  (NM_032444.2) | c.590T>C p.(Val197Ala) | rs147826749 (<0.01) | missense (0)^╪^ | 1 |
| 65 |  | c.753G>A  p.(=) | rs8061528 (0.25) | synonym | 1 |
| 66 |  | c.4580C>T p.(Pro1527Leu) | rs149362820 (nr) | missense (0)^╪^ | 1 |
| 67 |  | c.5501A>G p.(Asn1834Ser) | rs111738042 (<0.01) | missense (0)^╪^ | 1 |
| Total TP variants | | | | | 83 |

^┼^TP (true positive) variants are all heterozygous except when indicated for mutation#34. Nucleotide A of the ATG translation initiation start site of the cDNAs from reference sequence is indicated as nucleotide +1. In gray, pathogenetic variants of the disease-causing gene. In darker gray, heterozygous potential pathogenetic variants in a gene different from that causing the disease.

^╪^Effect of the missense variations was evaluated using four pathogenicity prediction programs, such as PoliPhen-2 ([http://genetics.bwh.harvard.edu/pph2/](https://posta.um.fvg.it/owa/UrlBlockedError.aspx)), Mutation Taster (http://www.mutationtaster.org/) Mutation Assessor (http://mutationassessor.org/), and SIFT (<http://sift.jcvi.org>). Since the different software use different prediction scales, we converted each output assigning a value from 0 to 2. Specifically, we assigned values 0, 1, and 2 to the PolyPhen-2 prediction of "benign", "possibly damaging", and "probably damaging", respectively. Values of 0 and 2 were attributed to "polymorphism" and "disease causing" output of Mutation Taster, respectively. To the Mutation Assessor predictions of "neutral", "low", and "medium" we assigned values of 0, 1, and 2, respectively. Finally, when the SIFT output was "tolerated" the value was 0 and for "not tolerated" was 2. The pathogenicity score was obtained by summing the values attributed to the output from the single software. Scores ≥5 were considered pathogenetic. The in silico analyses for detection of potential splicing mutations were carried out using Human Splicing Finder Version 2.4.1 (http://www.umd.be/HSF/).

^§^Confirmed by RT-PCR by De Rocco at al. (2014)

**Table 4S. False positive (FP) variants identified during characterization of novel FA alleles**

| Number | Gene | FP variants^┼^ | rs number  (MAF in 1000 Genome Database) | Status | N. of samples with FP | Sequence contest of FP^╪^ |
| --- | --- | --- | --- | --- | --- | --- |
| 1 | *FANCC* (NM_000136.2) | c.1297del | nr | homozygous | 6 | GCCCC**C**GTGATGGG |
| 2 | *FANCD2* (NM_001018115.1) | c.696-19C>T | nr | heterozygous | 1 | CTTTTTT**C**TTTTTCT |
| 3 |  | c.1134+39T>G | nr | heterozygous | 1 | CAGAC**T**TAAAAGTA |
| 4 |  | c.1170C>T | rs1122887807 (nr) | heterozygous | 25 | TAG**C**ACCAATACTCAGACAAA |
| 5 |  | c.1179T>C | rs72492998 (nr) | heterozygous | 25 | TAGCACCAATAC**T**CAGACAAA |
| 6 |  | c.1214A>G | rs73126218 (nr) | heterozygous | 25 | CTAAGAA**A**TAAGAT |
| 7 |  | c.2715+96A>T | nr | heterozygous | 1 | GAAAAAAAA**A**TTAG |
| 8 | *FANCE* (NM_021922.2) | c.970-26T>G | rs192832780 (0.0009) | heterozygous | 1 | TCCCGGTGTCC**T**CTCTCCCCCC |
| 9 |  | c.1113+23_1113+24insC | nr (0.0018) | heterozygous | 1 | TGGGAGGTACTC(**C)**AGAGTGCCAAG |
| 10 | *FANCI* (NM_001113378.1) | c.3537+41C>A | nr | heterozygous | 1 | TTCTAC**C**CCAGT |
| 11 |  | c.3947G>A | rs138461165 (0.0005) | heterozygous | 1 | ATGGGG**G**ACAGAA |
| 12 | *FANCJ* (NM_032043.2) | c.1543G>T | nr | heterozygous | 2 | GCAAGA**G**AAGTA |
| 13 |  | c.2575+8C>A | nr | heterozygous | 22 | GAT**C**TCAGCTGGG |
| 14 | *FANCL* (NM_018062.3) | c.375-26A>T | nr | heterozygous | 3 | GATC**A**TTTTTTATTC |
| 15 |  | c.375-25T>A | nr | heterozygous | 4 | GATCA**T**TTTTTATTC |
| 16 | *FANCM* (NM_020937.2) | c.1397-16_1397-14del | nr | homozygous | 1 | TTAAAGTTT**TTA**TATATATATATAG |
| 17 | *FANCP*  (NM_032444.2) | c.3661del | nr | homoztgous/ heterozygous | 2 | GAGGGG**G**CGCTGCC |
| 18 |  | c.4259C>A | nr | heterozygous | 1 | GACAGTGACCCCC**C**AATTCCAATTGAC |
| 19 |  | c.4261A>C | nr | heterozygous | 1 | GACAGTGACCCCCCA**A**TTCCAATTGAC |
| Total FP annotations | | | | | 124 |  |

^┼^ Nucleotide A of the ATG translation initiation start site of the cDNAs from reference gene sequence is indicated as nucleotide +1.

^╪^ In bold are the nucleotide changes.

**Table 5S. Mann-Whitney test between males and females***

| Sample | Gender | *P*  (*FANCB* sample / *FANCB* ♀ control) | *P*  (*FANCB* sample / *FANCB* ♂ control) |
| --- | --- | --- | --- |
| All males | ♂ | <.0001 | 1 |
| All females | ♀ | 1 | <.0001 |
| P12 | ♂ | <.0001 | .0926 |
| P26 | ♂ | <.0001 | .9992 |
| P14 | ♂ | <.0001 | .3974 |
| P15 | ♂ | <.0001 | .8992 |
| P13 | ♂ | <.0001 | 1 |
| P25 | ♂ | <.0001 | .9237 |
| P7 | ♂ | <.0001 | .7416 |
| W1 | ♂ | <.0001 | .746 |
| P5 | ♂ | <.0001 | .7212 |
| P8 | ♂ | <.0001 | .6867 |
| P20 | ♂ | <.0001 | .8686 |
| P30 | ♂ | <.0001 | .3058 |
| **P16** | **♂** | **<.0001** | **.0477** |
| **P28** | **♀** | **.016** | **<.0001** |
| P10 | ♀ | .1654 | <.0001 |
| P11 | ♀ | .0902 | <.0001 |
| P18 | ♀ | .3932 | <.0001 |
| P6 | ♀ | .2542 | <.0001 |
| P21 | ♀ | .8961 | <.0001 |
| P27 | ♀ | .9754 | <.0001 |
| W2 | ♀ | .9187 | <.0001 |
| P24 | ♀ | .7117 | <.0001 |
| P4 | ♀ | .5231 | .0011 |
| P23 | ♀ | .4632 | <.0001 |
| P9 | ♀ | .1809 | <.0001 |
| P29 | ♀ | .7117 | <.0001 |
| P19 | ♀ | .7958 | <.0001 |
| P17 | ♀ | .0775 | <.0001 |
| **P22** | **♀** | **.0001** | **<.0001** |

*To exclude that the difference observed between males and females was due to random variations of the *FANCB* coverage, the Mann-Whitney test (distribution of the data was non-normal) was performed. Samples were split in two control groups (males and females) against which we compared all the intersample normalisation ratios of *FANCB* amplicons of each patient. The difference between median of all patients and the median of controls of the opposite gender was statistically significant, while the difference between median of all patients and the median of true gender was not significant, except in samples P16, P22, and P28. Male P16 has a significantly higher median (median=0.56; IQR=0.52-0.62) than the median of male population (median=0.52; IQR=0.44-0.62). Females P22 and P28 had a significantly higher (median=1.28; IQR=1.16-1.41) and lower (median=0.91; IQR=0.78-1.13) median than that of the female population (median=1.05; IQR=0.90-1.24) (Figure 2A).

**Supplemental figures**

**Figure 1S**

**
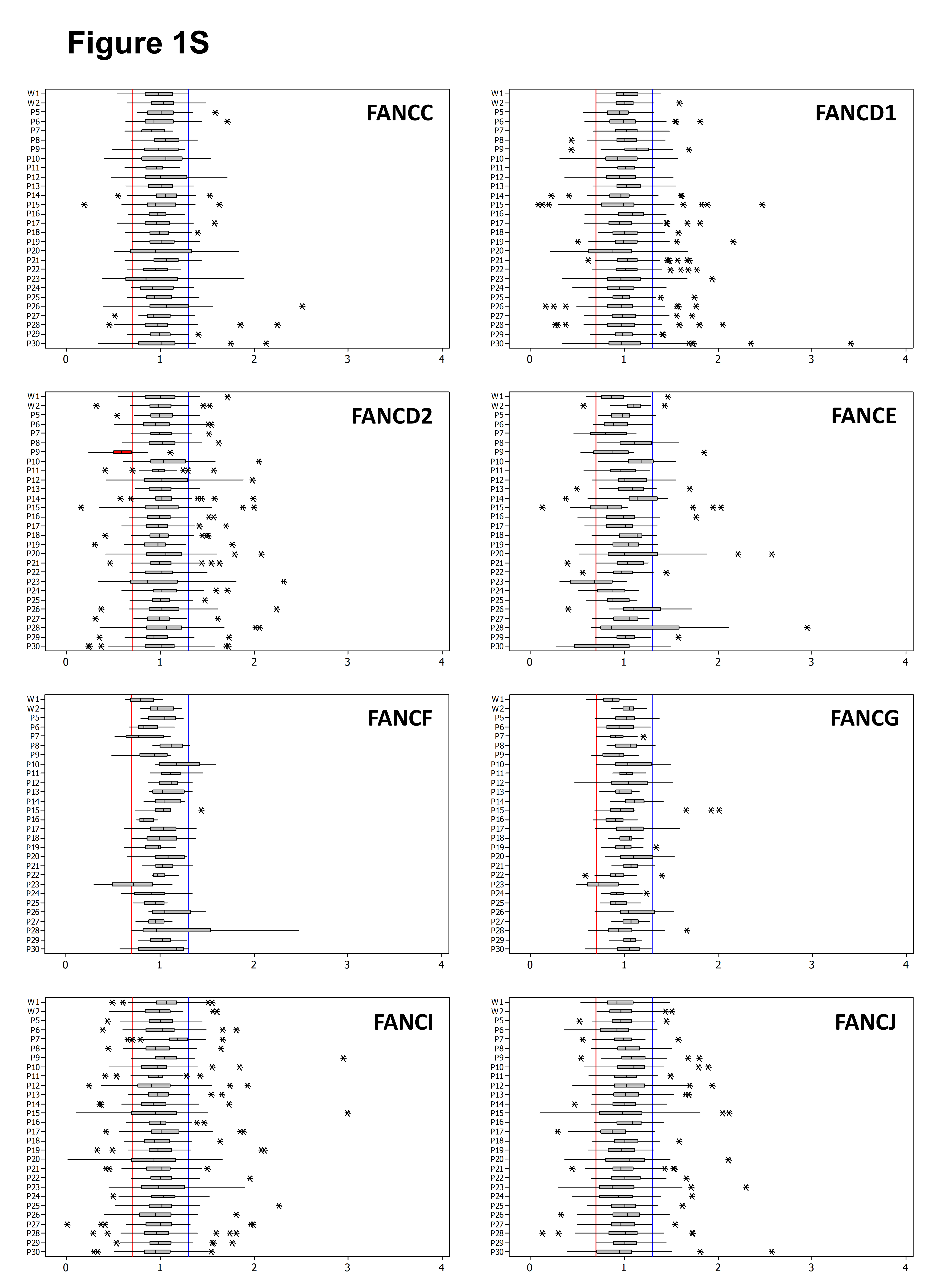
**

**
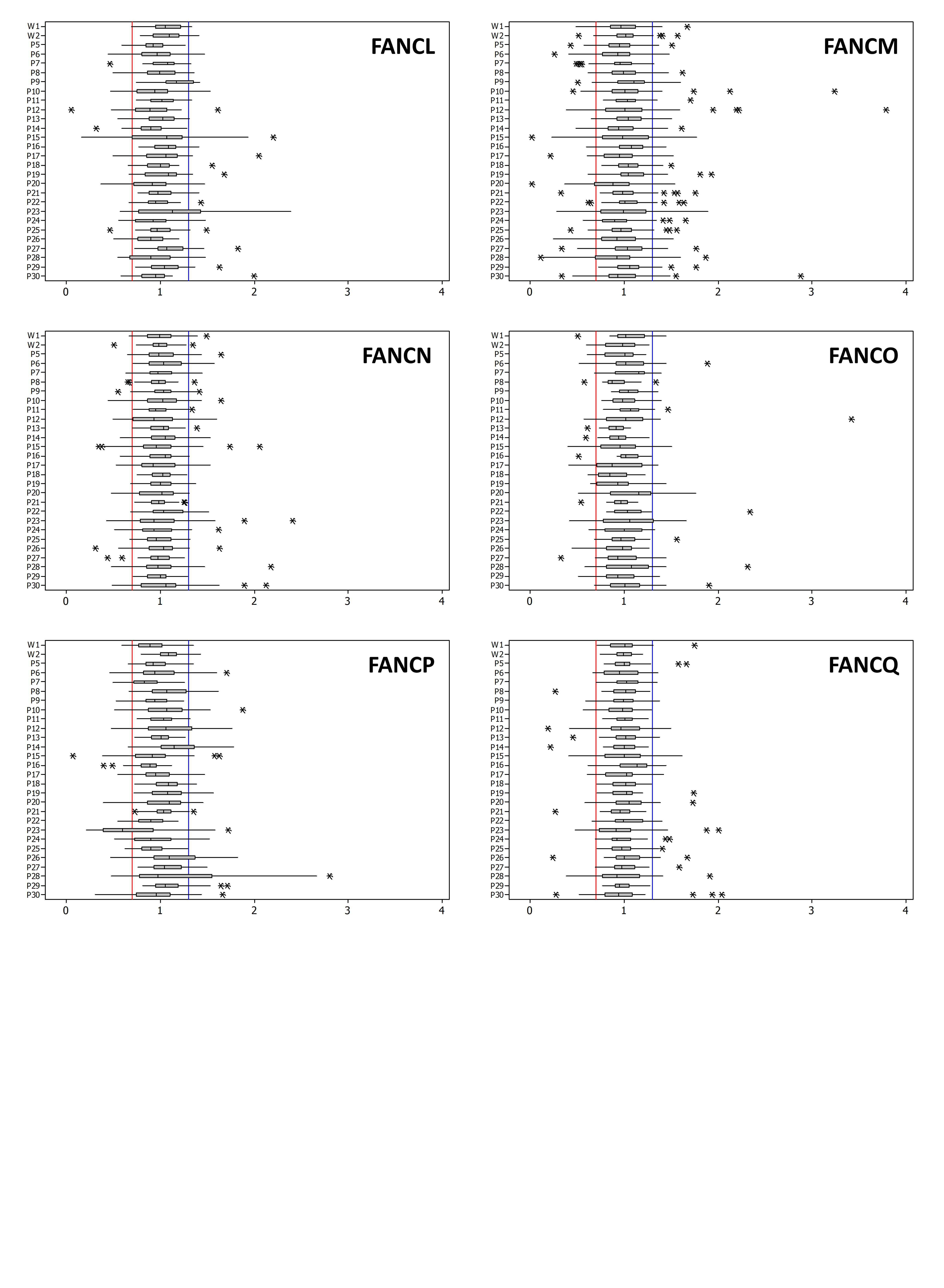
**

**Figure 1S. CNV analysis of the FA genes**. The analysis is shown for all the FA genes except for *FANCA* and *FANCB* (Fig. 1) in 28 of the 30 samples included in this study (samples P3 and P24 have been excluded for their low amplicon uniformity). Box plots report median, interquartile range (IQR) and outliers (asterisks). A median below 0.7 or between 0.7 and 1.3 (red and blue vertical lines) is indicative of one or two copies, respectively of the gene. The median is in the normal range for all samples except for P9 in *FANCD2* and P23 in both *FANCE* and *FANCP*. The 1^st^ and the 3^rd^ quartiles are low and above the threshold of 0.7 and 1.3 in several samples, but for most of which with only slight deviations. Of note, *FANCE* and *FANCF*, the two genes covered by the lowest number of amplicons (18 and 8, respectively), show IQR deviations in a higher number of samples. Since in sample P23 both median and IQR were outside the normal ranges in eight genes without knowing the reasons, the deviations were not considered reliable enough for suspecting any CNV.

**Figure 2S**


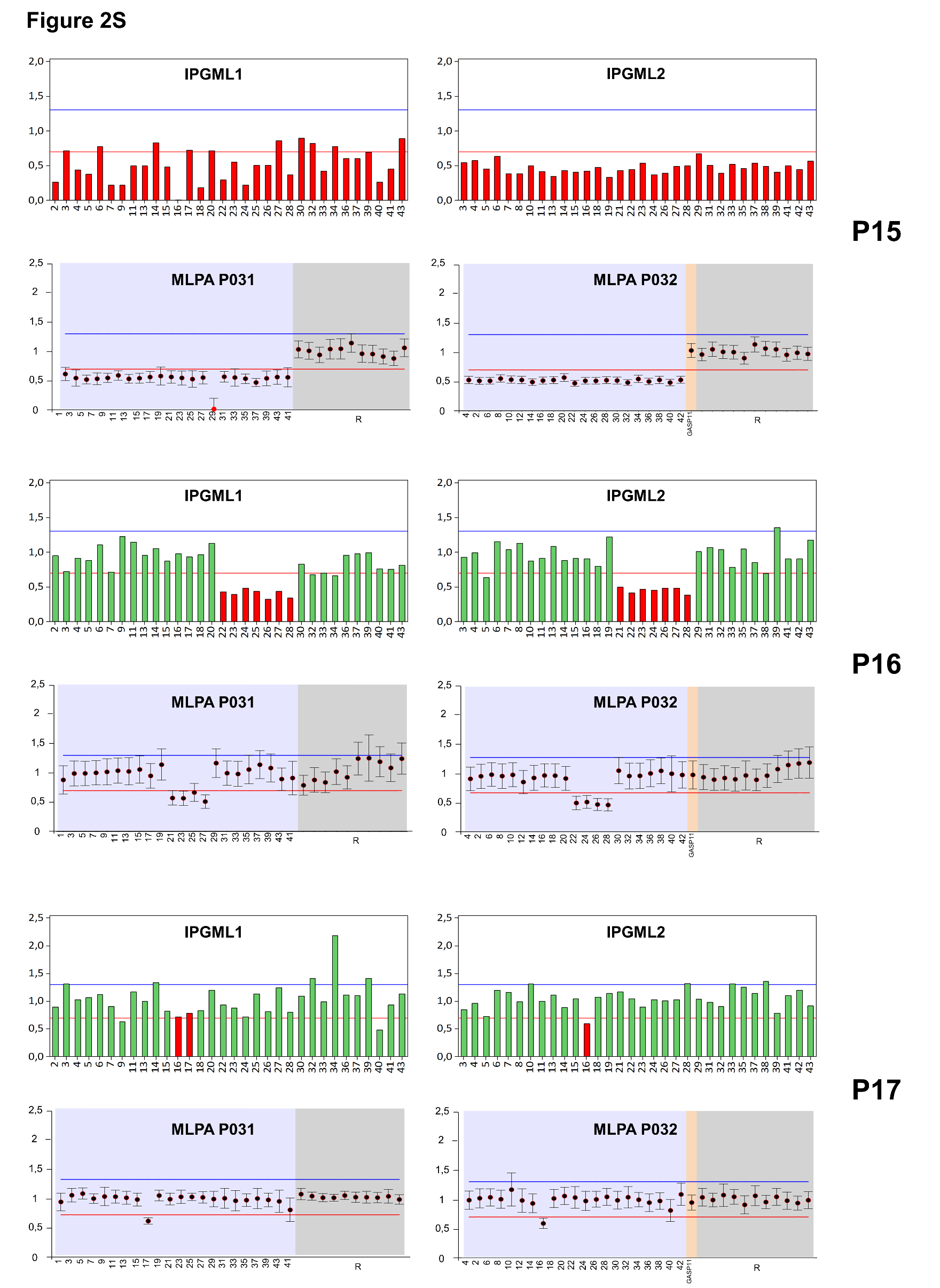


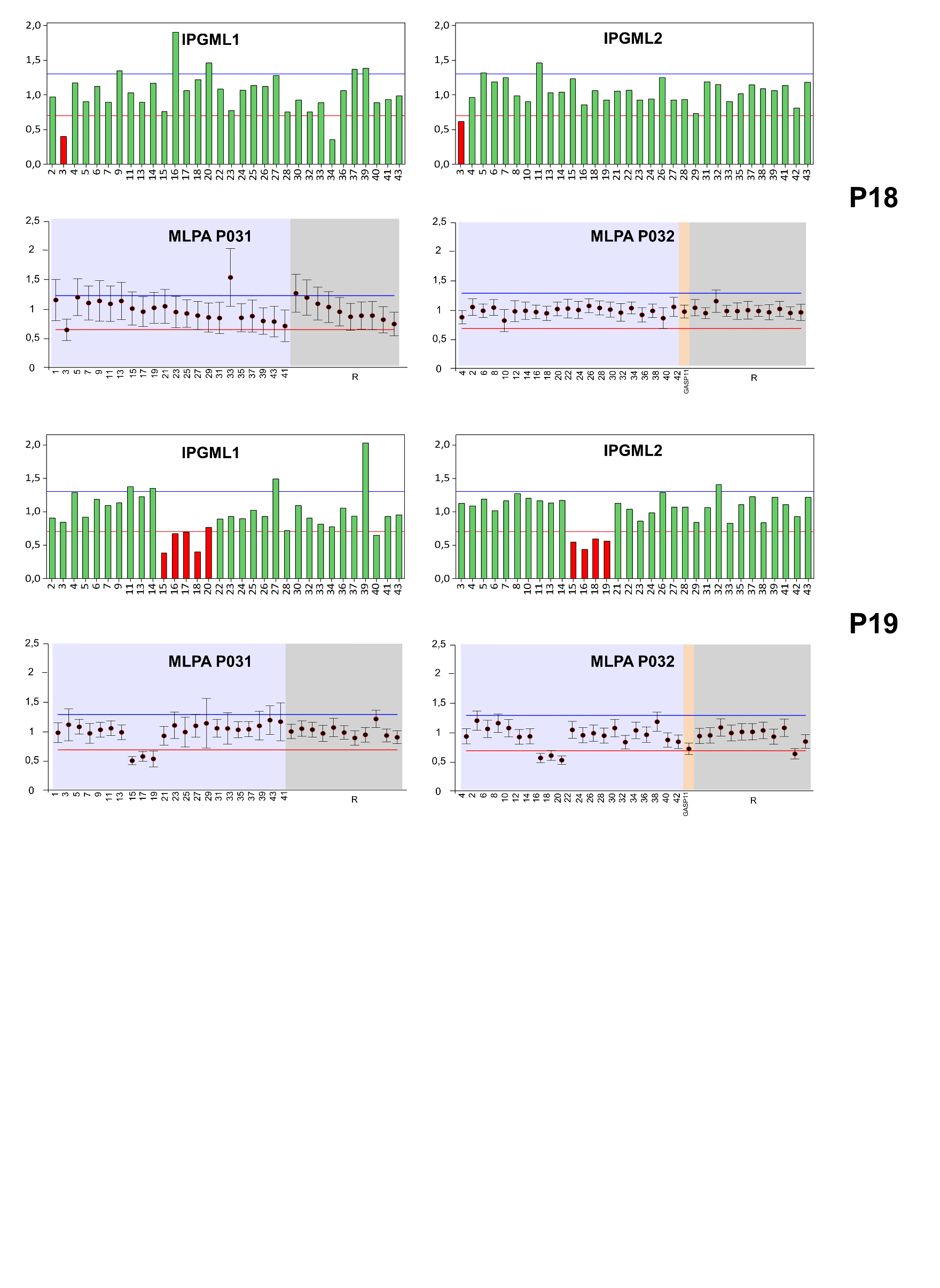


**Figure 2S. Detection of large intragenic known deletions of *FANCA*.** IPGM and MLPA analysis showing deletions of the entire gene (P15), exons 21-28 (P16), exons 16-17 (P17), exons 3 (P18), and exons 15-20 (P19). Amplicons from the two IPGM libraries (IPGML1 and IPGML2) are reported in graphs showing hemizygous amplicons in red. MLPA output of two probes mix (MLPAP031 and MLPAP032) from the Coffalayzer.net software, showing *FANCA* exons and reference loci (R) values. In both IPGM and MLPA analysis, the intersample normalization of deleted adjacent exons is under the threshold of 0.7 (red line). Of note, in graph MLPAP031 of P15, the apparent homozygous deletion of exon 29 is a false positive of MLPA because the mutation on the second allele (c.2812_2830dup) is localized within the probe annealing sequence of exon 29.

**Figure 3S**


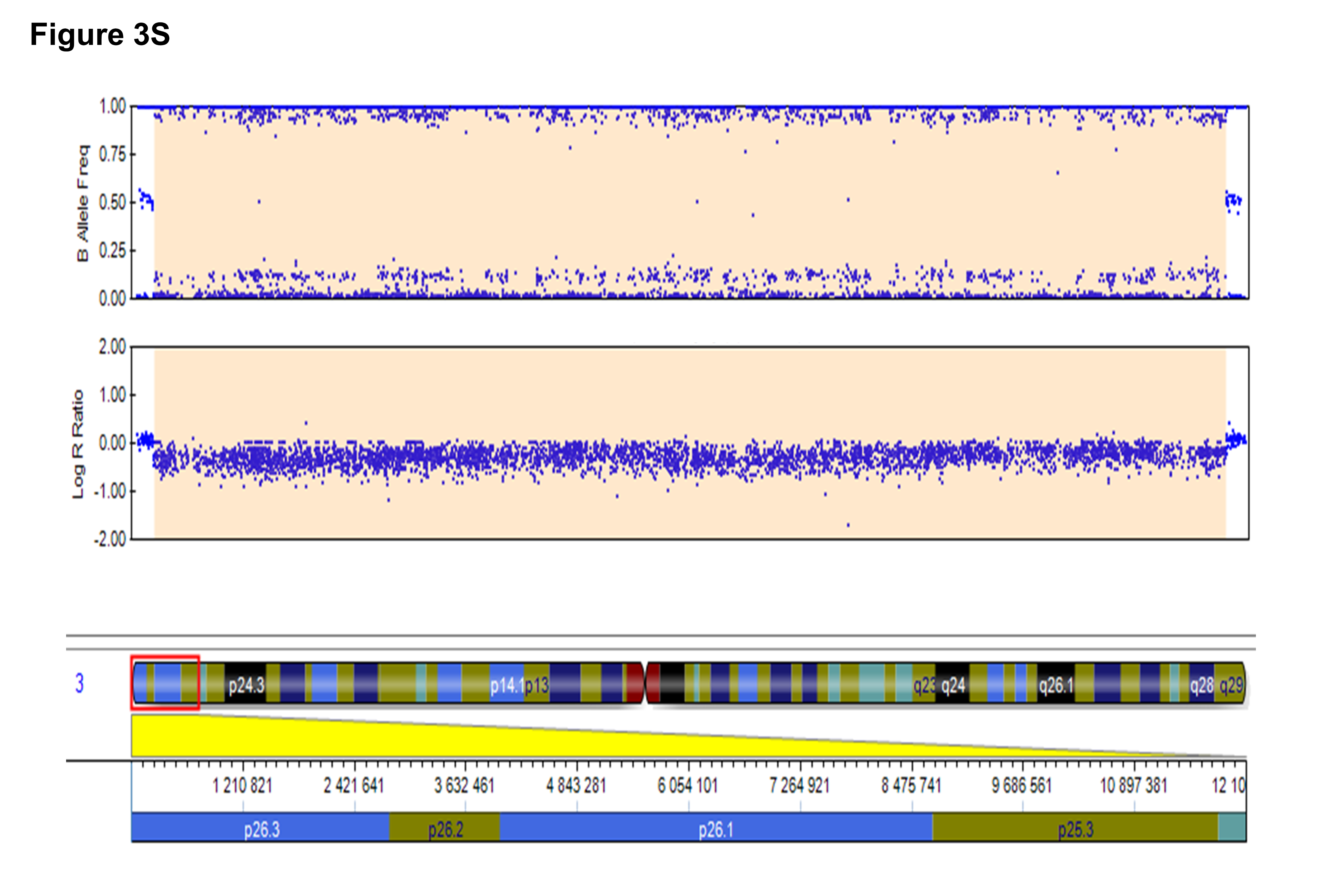


**Figure 3S. Graphical output for copy number using SNP-based arrays on chromosome 3 in P9**. The plot for the B allele frequency shows a heterozygous deletion of 11.6 Mb(235,748-11,880,816) on chromosome 3p26.3-p25.2 containing the *FANCD2* gene. In this patient the plot has 10% heterozygous (AB) SNP calls, as shown by the additional allele frequency, indicating a mosaicism of approximately 90%. For probes that are normal copy number, the signal intensity ratio of the subject versus controls is expected to be 1, and log_2_ R ratio should be approximately 0.0 (log_2_1 = 0). In the other plot loss of copy number results in a negative log_2_ ratio of approximately −0.5. SNP array analysis was performed using the Human OmniExpress-12 Bead Chip (Illumina Inc., San Diego, CA) according to Illumina’s Infinium HD Assay protocol. Normalization of raw image intensity data, genotype clustering and individual sample genotype calls were performed using Illumina’s GenomeStudio software v2011.1 (cnv partition 3.2.0). The CNV calls were determined with generalized genotyping methods implemented in the Penn CNV program.

**Figure 4S**

**
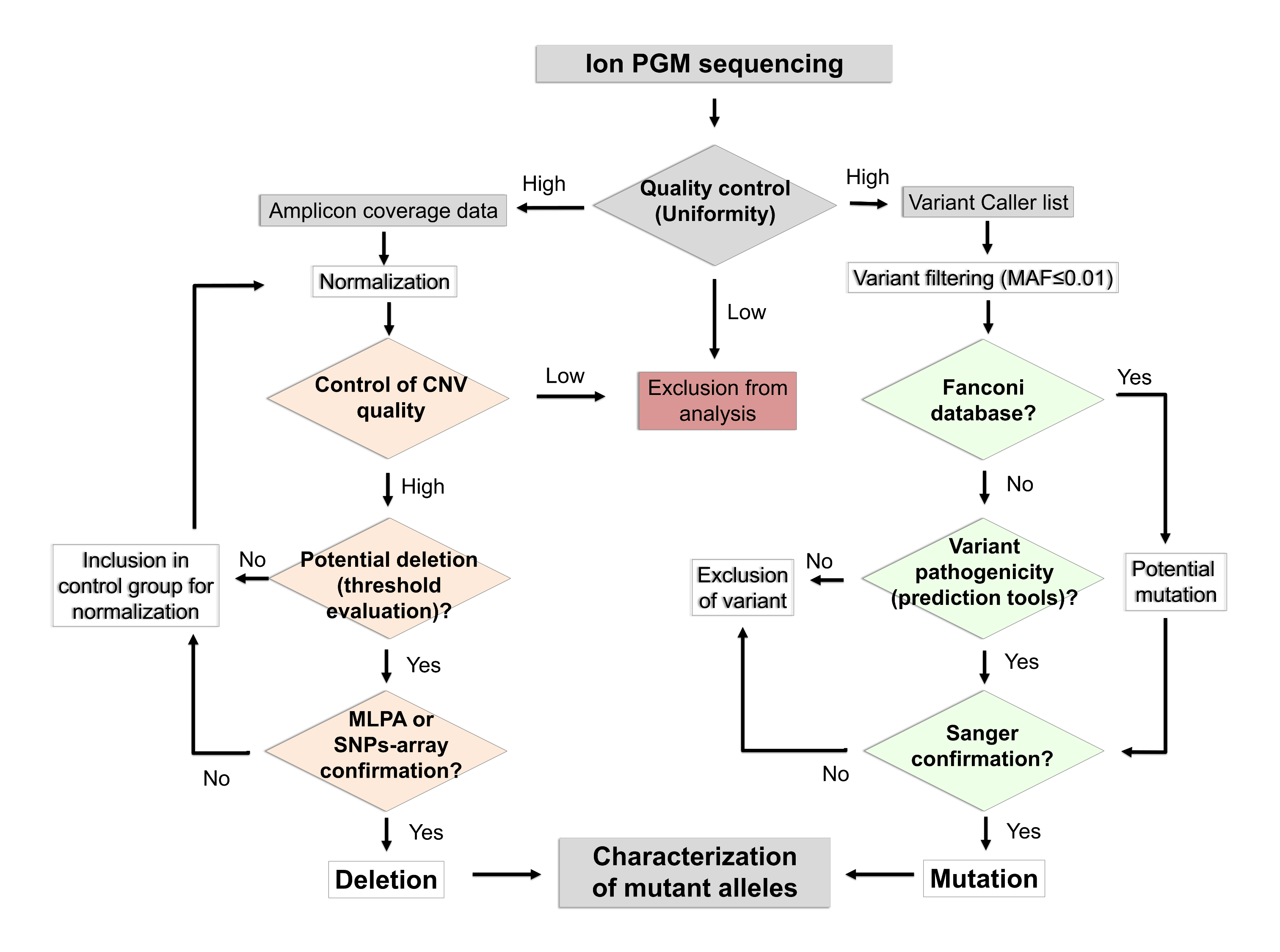
**

**Figure 4S. Schematic representation of the molecular diagnostic workflow that can be applied in FA using the IPGM sequencing.**
